# Efficient Transient Expression of Recombinant Proteins in Plants by the Novel pEff Vector Based on the Genome of Potato Virus X

**DOI:** 10.3389/fpls.2017.00247

**Published:** 2017-02-28

**Authors:** Eugenia S. Mardanova, Elena A. Blokhina, Liudmila M. Tsybalova, Hadrien Peyret, George P. Lomonossoff, Nikolai V. Ravin

**Affiliations:** ^1^Institute of Bioengineering, Research Center of Biotechnology of the Russian Academy of SciencesMoscow, Russia; ^2^Research Institute of InfluenzaSt. Petersburg, Russia; ^3^Department of Biological Chemistry, John Innes CentreNorwich, UK

**Keywords:** transient expression, viral vector, *Agrobacterium*, potato virus X, virus-like particle

## Abstract

Agroinfiltration of plant leaves with binary vectors carrying a gene of interest within a plant viral vector is a rapid and efficient method for protein production in plants. Previously, we constructed a self-replicating vector, pA7248AMV, based on the genetic elements of potato virus X (PVX), and have shown that this vector can be used for the expression of recombinant proteins in *Nicotiana benthamiana*. However, this vector is almost 18 kb long and therefore not convenient for genetic manipulation. Furthermore, for efficient expression of the target protein it should be co-agroinfiltrated with an additional binary vector expressing a suppressor of post-transcriptional gene silencing. Here, we improved this expression system by creating the novel pEff vector. Its backbone is about 5 kb shorter than the original vector and it contains an expression cassette for the silencing suppressor, P24, from grapevine leafroll-associated virus-2 alongside PVX genetic elements, thus eliminating the need of co-agroinfiltration. The pEff vector provides green fluorescent protein expression levels of up to 30% of total soluble protein. The novel vector was used for expression of the influenza vaccine candidate, M2eHBc, consisting of an extracellular domain of influenza virus M2 protein (M2e) fused to hepatitis B core antigen. Using the pEff system, M2eHBc was expressed to 5–10% of total soluble protein, several times higher than with original pA7248AMV vector. Plant-produced M2eHBc formed virus-like particles *in vivo*, as required for its use as a vaccine. The new self-replicating pEff vector could be used for fast and efficient production of various recombinant proteins in plants.

## Introduction

Recombinant proteins, including those for medical purposes, can be produced in different expression systems, including bacteria, yeast, plants, and mammalian cells. Recently, plants have become a promising system for protein production. The advantages of using plants for protein production are the low cost of cultivation of plants and the safety of products due to the absence of pathogens common to both plants and animals.

Recombinant proteins can be produced in plants by genetic transformation or transient expression (Pogue et al., 2002; Gleba et al., 2007; Lico et al., 2008). The creation of a transgenic plant line and, consequently, production of a protein from these plants requires considerable time and cost. The level of expression of target proteins by transgenic plants is usually low, leading to a high cost of products due to the difficulty of their purification (Edelbaum et al., 1992; Kusnadi et al., 1997). Transient gene expression provides a rapid alternative to the generation of stably transformed plants (Kapila et al., 1997). The advantages of such plant biofactories are the ease of manipulation, speed, low cost, and usually higher protein yield per weight of plant tissue.

Taking advantage of the ability of *Agrobacterium tumefaciens* to transfer a defined segment of DNA (T-DNA) to the plant nucleus (Gelvin, 2003), the molecular genetic modification of plants is most often performed using binary vector systems. These vectors consist of two parts: the first component is the T-DNA-containing cassette which is transferred to the plant cell; the second component is the vector backbone, which carries plasmid replication functions for both *Escherichia coli* and *A. tumefaciens*, selectable marker genes for bacteria and, optionally, genes encoding plasmid mobilization functions (Komori et al., 2007; Lee and Gelvin, 2008). The plant hosts *Lactuca sativa, Arabidopsis thaliana*, or *Nicotiana tabacum* have been used, but more recently, *Nicotiana benthamiana* has become the preferred experimental plant host (Goodin et al., 2008).

A promising area in the creation of vector systems for expression in plants involves the exploitation of sequences from self-replicating viruses. Several different plant viruses have been used as the basis for self-replicating viral vectors: tobacco mosaic virus (TMV) (Marillonnet et al., 2004; Chapman, 2008; Dohi et al., 2008), potato X virus (PVX) (Baulcombe et al., 1995; Komarova et al., 2006), cowpea mosaic virus (CPMV) (Sainsbury et al., 2009a), tobacco etch virus (TEV) (Dolja et al., 1992), as well as several others. Such vectors can be placed within the T-DNA portion of a binary plasmid providing a means for the transfer of the vector into plant cells during infiltration of leaf tissue with *Agrobacterium* suspensions (Marillonnet et al., 2005). Plant viral vectors can provide sufficient amounts of the desired protein, up to 1 g/kg of plant biomass (Gleba et al., 2007; Thuenemann et al., 2013), to allow detailed immunological characterization.

There are many factors influencing the yield of proteins produced in plants using viral expression systems: promoter activity, gene silencing, translation efficiency, vector size, *etc*. Post-transcriptional gene silencing (PTGS) is one of the reasons of low level expression of recombinant proteins in agroinfiltrated leaves. The use of suppressors of gene silencing, such as the P19 protein from Tomato bushy stunt virus (TBSV), to reduce PTGS has had an enormous impact on yields (Peyret and Lomonossoff, 2015). Likewise, P24 protein from grapevine leafroll-associated virus-2 (GRLaV2) has been identified as a strong silencing suppressor that is capable of preventing induction of silencing by double-stranded inverted repeats in plants (Chiba et al., 2006).

To improve and simplify experiments on recombinant protein production in plants, a special series of non-replicating viral vectors, named pEAQ, was developed (Sainsbury et al., 2009b). These vectors contain a modified 5′-untranslated region (UTR) and the 3′-UTR from CPMV RNA-2 (CPMV-*HT*) within the T-DNA region of binary vector pBINPLUS. Moreover, more than 7 kb of non-essential sequence was removed from the pBINPLUS backbone; this reduction in size has facilitated cloning procedures. The vector pEAQspecialK-GFP-HT additionally contains an expression cassette for the P19 silencing suppressor and a neomycin phosphortransferase (NPT) II cassette conferring resistance to kanamycin.

Previously, we constructed a viral vector, pA7248AMV-GFP (Mardanova et al., 2009), based on genetic elements from PVX and have shown that insertion of the 5′-UTR from RNA 4 of alfalfa mosaic virus (AMV), previously demonstrated to be a translational enhancer (Jobling and Gehrke, 1987), immediately upstream of the target gene increases the production of the target protein by the recipient plant three- to fourfold. This vector was successfully used for the expression of various vaccine candidate proteins in *N. benthamiana* (Ravin et al., 2012; Mardanova et al., 2015, 2016).

In this work we combine the advantages of pEAQ vectors, PVX-based vectors, and P24 silencing suppressor to create a new vector for quick and efficient transient expression of recombinant proteins in plants.

## Materials and Methods

### Media and Reagents

Bacteria were grown in LB broth or on LB agar plates at 37°C (*E. coli*) or at 28°C (*A. tumefaciens*). If necessary, the media were supplemented with kanamycin (50 μg/ml), rifampicin (50 μg/ml), or gentamycin (25 μg/ml).

### Synthetic Nucleotide Sequences

The following synthetic oligonucleotides were used: BlnI_35S_F (ATCCTAGGGTCAACATGGTGGAGCACGA), Nos-T_SnaBI_R (ATTACGTAGATCTAGTAACATAGATGACACCGCG), XhoI_35S_F (ATCTCGAGGTCAACATGGTGGAGCACGA), Nos-T_Bst1107I_R (TAGTATACGATCTAGTAACATAGATGACACCGCG), P19-BHI-F (ATGGATCCATGGAACGagCTATACAA), P19-SwI-R (ATATTTAAATTTACTCGCTTTCTTTTTCGAAGG).

The sequence of the P24 gene was codon-optimized *in silico* for expression in *N. benthamiana* and synthesized *in vitro* (Evrogen, Russia). Additionally, this sequence contains *Bam*HI, *Bsr*GI, and *Swa*I restriction sites for cloning procedures (Additional file 1: Supplementary Figure S1).

### Vector Construction

As a backbone for construction of expression vectors we used plasmid pEAQselectK (GenBank: GQ497231.1), in which the backbone region is shorter than in pBINPLUS (Sainsbury et al., 2009b). To make this vector suitable for subsequent cloning steps, we first removed restriction sites *Asc*I and *Sma*I from its multiple cloning site. DNA of pEAQselectK was treated with *Asc*I and *Sma*I, then the ends were made blunt using Klenow fragment and the DNA was circularized by self-ligation. The resulting plasmid was named pEf.

A synthetic sequence of the P24 gene was cloned in plasmid pNRGFP (Mardanova et al., 2007) instead of the *gfp* gene using *Bam*HI and *Bsr*GI restriction sites. pNRGFP is a binary vector containing 35S promoter – *gfp* – NosT terminator expression cassette within the T-DNA region. Thereafter, the 35S – P24 – NosT fragment was amplified by PCR using primers BlnI_35S_F and Nos-T_SnaBI_R, and cloned in the pEf vector at *Sna*BI and *Bln*I restriction sites. The resultant plasmid was named pEf-p24.

A similar expression vector, pEf-p19, containing the gene encoding the silencing suppressor P19 from TBSV was also constructed. The P19 coding sequence was amplified from pEAQspecialK-GFP-HT (Sainsbury et al., 2009b) using primers P19-BHI-F and P19-SwI-R, and cloned into pEf-p24 instead of P24 using the *BamH*I and *Swa*I restriction sites.

**FIGURE 1:**
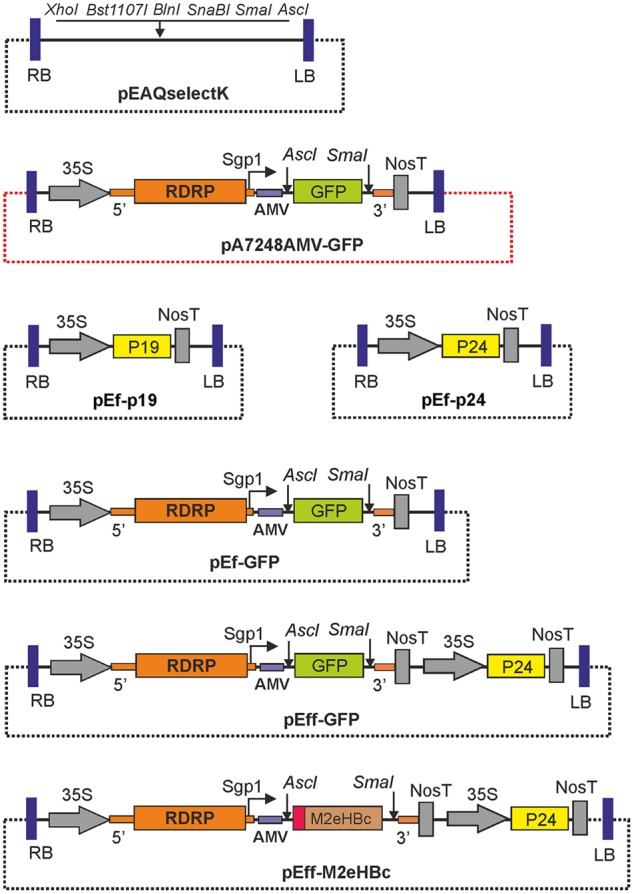
**Structures of the expression vectors.** RDRP, RNA-dependent RNA polymerase of PVX; Sgp1, subgenomic promoter of the PXV protein 25K; 5′ and 3′, the 5′-UTR and the 3′-UTR from PVX; AMV, the leader sequence of RNA 4 of the alfalfa mosaic virus; 35S, promoter of the cauliflower mosaic virus RNA; nosT, terminator of the nopaline synthase gene from *Agrobacterium tumefaciens*; P19, gene of silencing suppressor P19 from TBSV; P24, gene of silencing suppressor P24 from GRLaV2; GFP, green fluorescent protein gene; LB and RB are the left and right borders of tDNA. The backbone regions of vectors are shown by dotted lines. Note that all vectors contain the *nptII* genes within their T-DNA regions, not shown on the maps.

Expression vector pA7248AMV-GFP (Mardanova et al., 2009) contains the 5′- UTR of the PVX genome, the gene for viral RNA polymerase (RDRP), the subgenomic promoter of the gene for PVX 25K transport protein (Sgp1), the reporter *gfp* gene downstream of the AMV leader sequence, and the 3′-UTR of the PVX genome. This sequence is inserted between the 35S promoter and NosT terminator within the T-DNA region of pBIN19 binary vector. Unique restriction sites *Asc*I and *Sma*I located at 5′ and 3′ ends of *gfp* can be used for cloning of a target gene to replace *gfp* (**Figure 1**). The sequence comprising the whole expression cassette between the 35S promoter and NosT was amplified by PCR using pA7248AMV-GFP DNA as a template and primers XhoI_35S_F and Nos-T_Bst1107I_R. Cloning of this fragment at *Xho*I and *Bst*1107I restriction sites in pEf resulted in expression vector pEf-GFP. The same sequence was cloned into pEf-p24 at *Xho*I and *Bst*1107I restriction sites to make the final pEff-GFP vector. The nucleotide sequence of pEff-GFP vector has been deposited in the GenBank database under accession no KY439904. Structures of expression vectors are shown in **Figure 1**.

Recombinant vector pEff-M2eHBc was constructed for expression of the hybrid protein M2eHBc consisting of an extracellular domain of M2 protein (M2e) of influenza virus strain A/Duck/Potsdam/1402-6/1986 fused to the N-terminus of hepatitis B core antigen (HBc). The plant-optimized synthetic M2eHBc gene was excised from vector pA7248amvM2epHBc (Ravin et al., 2012) and cloned into the pEff vector using the *Asc*I and *Sma*I restriction sites.

### Agroinfiltration of *Nicotiana benthamiana* Leaves

The recombinant binary vectors were transferred from *E. coli* into *A. tumefaciens* strain GV3101 using electroporation. The resulting agrobacterial strains were grown overnight with shaking at 28°C. The cells (1.5 ml) were collected by centrifugation for 5 min at 4000 *g*, and the precipitate was resuspended in 1.5 ml of buffer containing 10 mM MES (pH 5.5) and 10 mM MgSO_4_. Leaves of 4- to 6-week old *N. benthamiana* plants were injected with suspension of agrobacteria (adjusted by buffer to OD_600_ of about 0.2) using a syringe without a needle. In case of co-infiltration, OD_600_ of 0.2 for each agrobacterial strain were used.

### Protein Isolation from Agroinfiltrated Plants and Quantitative GFP Assay

Three agroinfiltrated leaves on a single *N. benthamiana* plant were used for measurements of green fluorescent protein (GFP) expression. The leaves were ground in an extraction buffer [0.4 M sucrose, 50 mM Tris pH 8.0, 10 mM KCl, 5 mM MgCI_2_, 10% (v/v) glycerol, 10 mM β-mercaptoethanol] to obtain a homogeneous suspension. The suspension was centrifuged at 14 000 *g* for 15 min, and the supernatant containing soluble proteins was collected. Total protein content was determined by the Bradford assay kit (Biorad) with bovine serum albumin as standard. Expression of GFP in leaves was visualized under UV illumination and assayed by SDS-PAGE in a 15% (w/v) gel. For comparative quantitative measurements, GFP was assayed fluorometrically. Protein extracts were dissolved in 2.5 ml of phosphate buffered saline (50 mM sodium phosphate, pH 7.0, 200 mM NaCl), and GFP amount was determined with a Fluorat-02 fluorometer (Lumex, Russia), excitation at 390 nm, emission at 510 nm. The background level was determined and subtracted. For each of three analyzed leaves we considered the most efficient expression system vector as a reference (1 relative unit of GFP) and estimated the relative levels of GFP in other samples. The results in diagrams are expressed as the mean value ± standard deviation.

### SDS-PAGE and Western-Blot Analysis of M2eHBc Protein Samples

Protein extracts isolated from plant tissues were mixed with SDS-PAGE sample buffer containing β-mercaptoethanol. The samples were boiled for 10 min and subjected to SDS-PAGE on 15% (w/v) gels. After electrophoresis, gels were either stained with Coomassie brilliant blue or the proteins were transferred from the gel onto a Hybond-P membrane (GE Healthcare, USA) by electroblotting. To prevent non-specific binding of antibodies with the membrane, it was treated with 5% (w/v) solution of dry milk in TBS-T (150 mM NaCI, 20mM Tris, 0.1% (v/v) Tween 20, pH 8.0) buffer. The membrane was probed with mouse monoclonal antibodies specific for the M2e peptide and then incubated with rabbit anti-mouse secondary antibodies conjugated with peroxidase. Specific immunoreactive proteins were detected using a Western Blot ECL Plus kit (GE Healthcare, USA). The intensity of the bands in stained gels and blots was determined using Nonlinear.Dynamics.TotalLab.TL120.v2009-NULL software.

### Isolation of M2eHBc Virus-Like Particles from Plants and Electron Microscopy Analysis

Virus-like particles, formed by M2eHBc protein *in vivo*, were isolated form the soluble protein preparations using the method described in detail by Peyret (2015). Briefly, infiltrated tissue 7 days post-infiltration was blended in a Waring blender with three volumes of 0.1 M sodium phosphate (pH 7.2), filtered over miracloth, and clarified by centrifugation. The supernatant was filtered using a 0.45 μm syringe filter, then ultracentrifuged through a double sucrose cushion. The VLPs were recovered from the bottom of the sucrose cushion, dialysed against PBS, then ultracentrifuged over a Nycodenz density gradient. The particles thus purified were then observed by transmission electron microscopy.

Particle preparations were placed on carbon-coated copper grids and stained with 2% (w/v) uranyl acetate. The grids were examined using an FEI Tecnai 20 transmission electron microscope.

## Results and Discussion

### Reduction of vector size enhances protein production

To create an efficient plant expression vector we combined the advantages of different systems, namely, a binary vector reduced in backbone size, a self-replicating PVX-based vector with translational enhancer, and various suppressors of PTGS. Vector pEAQselectK (Sainsbury et al., 2009b) was used as a backbone. This plasmid is only 6974-bp long, it contains *nptIII* and *trfA* genes outside of T-DNA region, and *nptII* within T-DNA along with the multiple cloning site.

The main expression cassette originated from the previously constructed PVX-based vector pA7248AMV-GFP (Mardanova et al., 2009). This vector contains downstream of the 35S promoter an incomplete copy of the PVX genome including the 5′ UTR, RDRP gene, the first promoter of subgenomic RNA Sgp1 and the 3′ UTR. Upon agroinfiltration of plant tissue and transfer of T-DNA into plant cells, the mRNA transcription from the 35S promoter results in synthesis of the viral vector RNA, the viral vector is replicated in the infected cells, the subgenomic RNA encoding the desired gene is synthesized at high level, and the target protein is expressed. Insertion of translational enhancer from 5′-UTR of RNA 4 of AMV immediately upstream of the start codon of the target gene results in additional elevation of protein production (Mardanova et al., 2009). However, this vector system has two drawbacks. The first is its length since it is based on the pBIN19 plasmid that is almost 12 kb long itself. The second is the absence of a cassette for the expression of silencing suppressors in pA7248AMV-GFP. Thus, an efficient expression of the target proteins in plants with this system requires co-infiltration of leaves with another strain of *A. tumefaciens* to express such suppressors.

**FIGURE 2:**
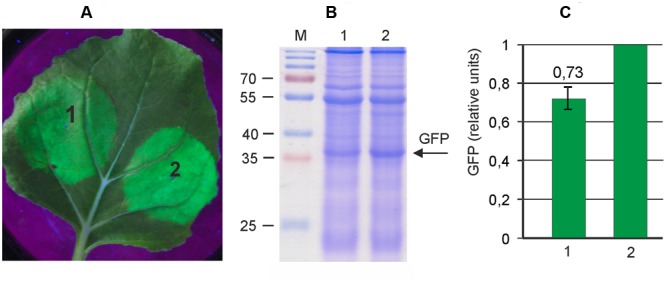
**Reduction of the size of an expression vector increases its efficiency.** Leaves were agroinfiltrated with either pA7248AMV-GFP (1) or pEf-GFP (2). **(A)** Visualization of GFP synthesized in agroinfiltrated *Nicotiana benthamiana* leaves. **(B)** SDS-PAGE analysis of proteins isolated from the agroinfiltrated zones of *N. benthamiana* leaves. The gel was stained with Coomassie brilliant blue. M, molecular weight marker (kD). **(C)** Fluorimetrically measured relative levels of GFP in protein samples.

Therefore, we first transferred the whole PVX-based expression cassette from pA7248AMV-GFP to pEAQselectK in order to minimize the size of vector. The resulting vector, pEf-GFP (**Figure 1**), is about 13 kb in contrast to pA7248AMV-GFP which is almost 18 kb. To compare the efficiencies of pEf-GFP and pA7248AMV-GFP, we transformed these vectors into *A. tumefaciens* strain GV3101, which was used for infiltration of leaves of *N. benthamiana* plants. Agroinfiltration zones for two vectors were located within one leaf. GFP synthesis was monitored by UV irradiation. The infiltrated plants were grown for 5 days, after which the leaves were collected and the soluble protein extracts were prepared. Synthesized GFP was assayed by SDS-PAGE, and quantified fluorometrically (**Figure 2**). The results show that the reduction in backbone size increases protein expression levels by about 40% in comparison to initial pA7248AMV-GFP vector. Thus, the pEf-GFP expression vector was used for subsequent vector improvements.

### Increased Efficiency of a Viral Vector able to Co-express Silencing Suppressor

Post-transcriptional gene silencing is known to be one of the most important factors limiting the efficiency of transient systems of recombinant protein expression in plants (Chicas and Macino, 2001). Although it had little negative effect on expression of GFP as shown above, this problem appeared to be more important for the expression of non-model proteins. For example, the use of pA7248AMV vectors for expression of influenza immunogens was only successful when the leaves were co-infiltrated with a silencing suppressor construct (Ravin et al., 2012; Mardanova et al., 2015).

**FIGURE 3:**
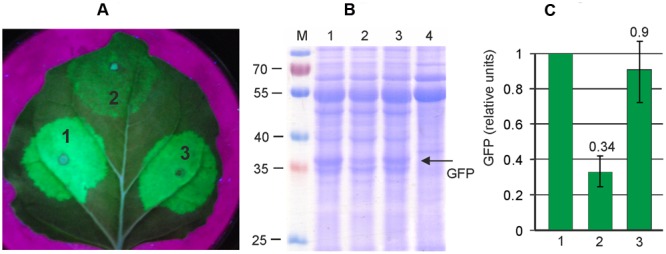
**Silencing suppressors increases the efficiency of viral expression system.** Leaves were agroinfiltrated simultaneously with pEf-GFP and pEf-p24 (1), with pEf-GFP alone (2), and simultaneously with pEf-GFP and pEf-p19 (3). **(A)** Visualization of GFP synthesized in agroinfiltrated *N. benthamiana* leaves. **(B)** SDS-PAGE analysis of proteins isolated from *N. benthamiana* leaves. The gel was stained with Coomassie brilliant blue. M, molecular weight marker (kD). Lane 4 show proteins isolated from non-infiltrated zone of the same *N. benthamiana* leaf. **(C)** Fluorimetrically measured relative levels of GFP in protein samples.

**FIGURE 4:**
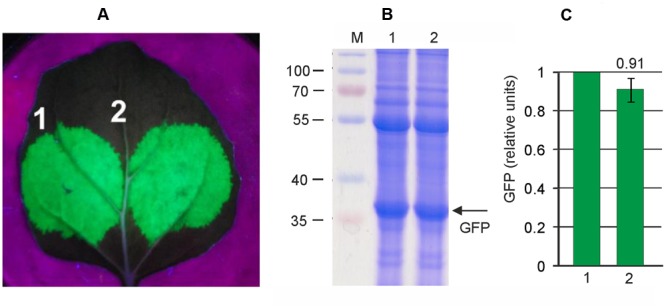
**High level production of GFP by the pEff expression system.** Leaves were agroinfiltrated simultaneously with pEf-GFP and pEf-p24 (1) or with pEff-GFP alone (2). **(A)** Visualization of GFP synthesized in agroinfiltrated *N. benthamiana* leaves. **(B)** SDS-PAGE analysis of proteins isolated form agroinfiltrated *N. benthamiana* leaves. The gel was stained with Coomassie brilliant blue. M, molecular weight marker (kD). **(C)** Fluorimetrically measured relative levels of GFP in protein samples.

Thus we aimed to insert a silencing suppressor cassette into the pEf-GFP vector. First we evaluated the efficiency of two known silencing suppressors, P24 from GRLaV2 and P19 from TBSV, in our self-replicating vector system. Both suppressor genes were cloned between the 35S promoter and nosT terminator, and these expression cassettes were inserted into the binary vector pEf, derived from pEAQselectK. We then compared GFP expression levels in *N. bethamiana* leaves infiltrated with (i) only pEf-GFP, (ii) simultaneously with pEf-GFP and pEf-p24, and (iii) simultaneously with pEf-GFP and pEf-p19. Protein samples were isolated 3 days after infiltration. The results show that both silencing suppressors increase GFP expression level about threefold, and that there is no significant difference between them (**Figure 3**). We chose the P24 for subsequent vector improvements. Thereafter we created the final vector pEff-GFP containing two expression cassettes. The first one contained 35S promoter, 5′-UTR of PVX, RDRP, Sgp1, *gfp*, 3′-UTR of PVX and Nos-T, and the second cassette contained 35S promoter, p24 and Nos-T. This vector eliminates the need to use co-infiltration, which simplifies manipulations. However, insertion of additional expression cassette in pEf-GFP increases the size of the vector which could have a negative effect on the performance of the expression system. We therefore compared the level of GFP production achieved with pEff-GFP vector alone and with levels obtained by co-infiltration of leaves with pEf-GFP and pEf-p24. GFP production was assayed 3 days after agroinfiltration. We found that GFP production level was similar for two variants (**Figure 4**). Under optimal conditions, the pEff vector provides a GFP expression level of about 30% of total soluble protein (**Figure 4**) which corresponds to 1 mg/g of fresh leaf tissue.

### Efficient Expression of Influenza Immunogen in *N. benthamiana* Using the pEff Vector

Green fluorescent protein is known as a model protein as it is stable and usually well-expressed in plant cells. Thus we examined the efficiency of the pEff system for production of an immunogen of potential clinical interest. We used the hybrid protein M2eHBc consisting of an extracellular domain of influenza virus M2 protein (M2e) fused to HBc at its N terminus. The hybrid protein M2eHBc formed virus-like particles carrying the M2e peptide on the surface and can be used as candidate influenza vaccine (Neirynck et al., 1999). Previously, we expressed M2eHBc protein in plants using the pA7248AMV vector. The protein was expressed up to 1–2% of total soluble protein in *N. benthamiana* leaves and was found to be able to form virus-like particles *in vivo* (Ravin et al., 2012). Immunization of mice with such plant-produced particles provided partial protection against the lethal influenza challenge (Ravin et al., 2012).

**FIGURE 5:**
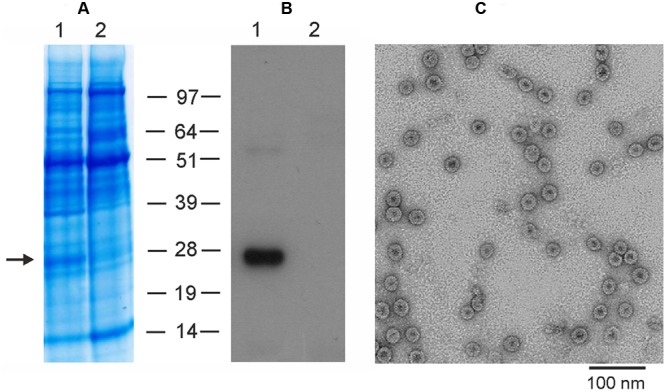
**Expression and purification of M2eHBc using the pEff vector.** Coomassie brilliant blue stained gel **(A)** and Western blot **(B)** of proteins isolated from *N. benthamiana* leaves and separated by SDS-PAGE. M, molecular weight marker (kD); (1) total soluble proteins isolated from leaves infiltrated with vector pEff-M2eHBc; (2) total soluble proteins isolated from uninfiltrated leaves. Antibodies against M2e were used in Western blotting. **(C)** Transmission electron microscopy of plant-produced M2eHBc virus-like particles. The grid was negatively stained with 2% (w/v) uranyl acetate and the scale bar is shown.

To produce M2eHBc particles in plants using the newly developed pEff vector, we cloned the M2eHBc sequence into this vector and performed agroinfiltration of *N. benthamiana* leaves. Protein samples were isolated 5 days after infiltration and analyzed by SDS-PAGE and Western-blotting (**Figure 5**). Our results show that the pEff expression system provides expression levels of M2eHBc of about 5–10% of total soluble protein, several times higher than obtained previously using the pA7248AMV vector or the non-replicating pEAQ-*HT* expression system (Thuenemann et al., 2013).

The recombinant M2eHBc particles were purified from agroinfiltrated plants by double sucrose cushion followed by a Nycodenz density gradient as described by Peyret (2015). The M2eHBc was assembled into spherical 30–35 nm particles which were similar to ones formed by the native HBc antigen, as revealed by transmission electron microscopy (**Figure 5**).

## Conclusion

In this study, by combination of a self-replicating expression cassette based on the elements of the potato virus X genome, a short binary vector and an additional expression cassette driving production of a suppressor of PTGS, we have significantly improved the PVX-based transient expression system. The use of the newly developed pEff system allowed high levels of expression of GFP in *N. benthamiana* plants to be achieved, at up to 1 mg/g of fresh leaf tissue without the need to co-infiltrate the leaves with another construct providing a silencing suppressor. We used this system to produce M2eHBc particles at up to 5–10% of total soluble protein, about 4–5 fold improvement over the original vector (Ravin et al., 2012). We propose that the new self-replicating pEff system could be used to produce various recombinant proteins in plants quickly and conveniently.

## Author Contributions

Conceived and designed the experiments: EM, GL, and NR. Performed the experiments and analyzed the data: EM, EB, LT, HP, and NR. Wrote the paper: EM, HP, GL, and NR.

## Conflict of Interest Statement

GL declares that he is a named inventor on granted patent WO 29087391 A1 which describes the pEAQ vector system used for the work described in this manuscript.

The other authors declare that the research was conducted in the absence of any commercial or financial relationships that could be construed as a potential conflict of interest.
