# Supplementary material for: Efficient Transient Expression of Recombinant Proteins in Plants by the Novel pEff Vector Based on the Genome of Potato Virus X
Source: Front Plant Sci. 2017 Feb 28;8:247. doi: 10.3389/fpls.2017.00247 (PMC5328947; doi:10.3389/fpls.2017.00247)
Supplement: Supplementary file 1 [file Image_1.PDF]

```
 BamHI
GGATCCGAAATGCGGGTGATAGTATCACCATACGAAGCTGAAGACATTTTAAAAAGATCAAC
AGACATGCTACGAAACATTGATAGCGGTGTTTTGTCTACAAAGGAATGCATTAAGGCTTTCA
GCACCATCACTAGAGACCTGCACTGTGCAAAGGCATCTTACCAATGGGGAGTTGACACAGGT
TTGTATCAACGTAATTGCGCAGAAAAGCACCTTATTGATACAGTTGAAAGTAACATAAGGTT
GGCACAGCCGCTTGTTAGGGAGAAGGTAGCAGTGCATTTTTGCAAGGATGAGCCTAAAGAAC
TTGTTGCCTTCATAACTAGAAAATATGTCGAATTGACTGGAGTAGGTGTTAGGGAAGCTGTT
AAAAGGGAGATGCGGAGTCTCACTAAGACAGTTCTCAATAAGATGTCCCTCGAAATGGCATT
CTATATGTCACCTAGAGCTTGGAAGAACGCAGAATGGCTGGAATTGAAATTTAGTCCCGTAA
AGATTTTCAGAGATTTGCTTCTCGATGTTGAAACTCTCAATGAACTCTGCGCAGAGGATGAC
GTTCATGTCGATAAAGTTAATGAAAACGGTGATGAGAACCATGATCTCGAACTTCAGGATGA
GTGCTGAATTTAAATTGTACA
           SwaI   BsrGI
```

Figure S1. Nucleotide sequence of the P24 gene optimized for expression in *Nicotiana benthamiana*.

The coding sequence is shaded. Restriction sites for *Bam*HI, *Bsr*GI, and *Swa*I enzymes are underlined
